# External laryngotracheal trauma: a case series and an algorithmic management strategy

**DOI:** 10.1007/s00405-024-08456-9

**Published:** 2024-01-23

**Authors:** Laurence Pincet, Gabriele Lecca, Ioanna Chrysogelou, Kishore Sandu

**Affiliations:** 1 Otorhinolaryngology & Head, Neck Surgery Department, Lausanne University Hospital, Centre Hospitalier Universitaire Vaudois, Rue du Bugnon 46, 1011 Lausanne, Switzerland; 2 Emergency Department, Hôpital Riviera Chablais, Rennaz, Switzerland

**Keywords:** Laryngotracheal injury, Airway management

## Abstract

**Objectives:**

External laryngotracheal trauma (ELT), blunt or penetrating, is a rare but potentially life-threatening injury. Immediate care in the emergency department can be challenging because it requires managing a potentially unstable airway and may have associated vascular injuries with massive bleeding. Here, we look at the details of injury, treatment measures, and outcomes in patients following ELT.

**Methods:**

We retrospectively analyzed 22 patients treated at our center for ELT from January 2005 up to December 2021 with varying grades of injury. We looked at their status at presentation, management strategy and functional status.

**Results:**

In our report, we include 18 men and 4 women having varying Schaefer injury grades. Eight patients had tracheostomy at presentation and eight had vocal fold immobility. Two patients were treated endoscopically, 12 had open surgery and 8 received no treatment. Of the patients undergoing open surgery, thyroid cartilage fracture was seen in 9 patients, thyroid plus cricoid fracture and cricotracheal separation were seen in 3 patients each. All patients were safely decannulated and spontaneous recovery of vocal cord palsy was seen in some patients.

**Conclusion:**

The success of managing ELT relies on fast decision-making, correct patient evaluation, securing the airway and maintaining the hemodynamic stability. Early surgical intervention must be aimed at optimally treating the larygotracheal injuries to prevent long-term disastrous consequences.

## Introduction

**Fig. 1:**
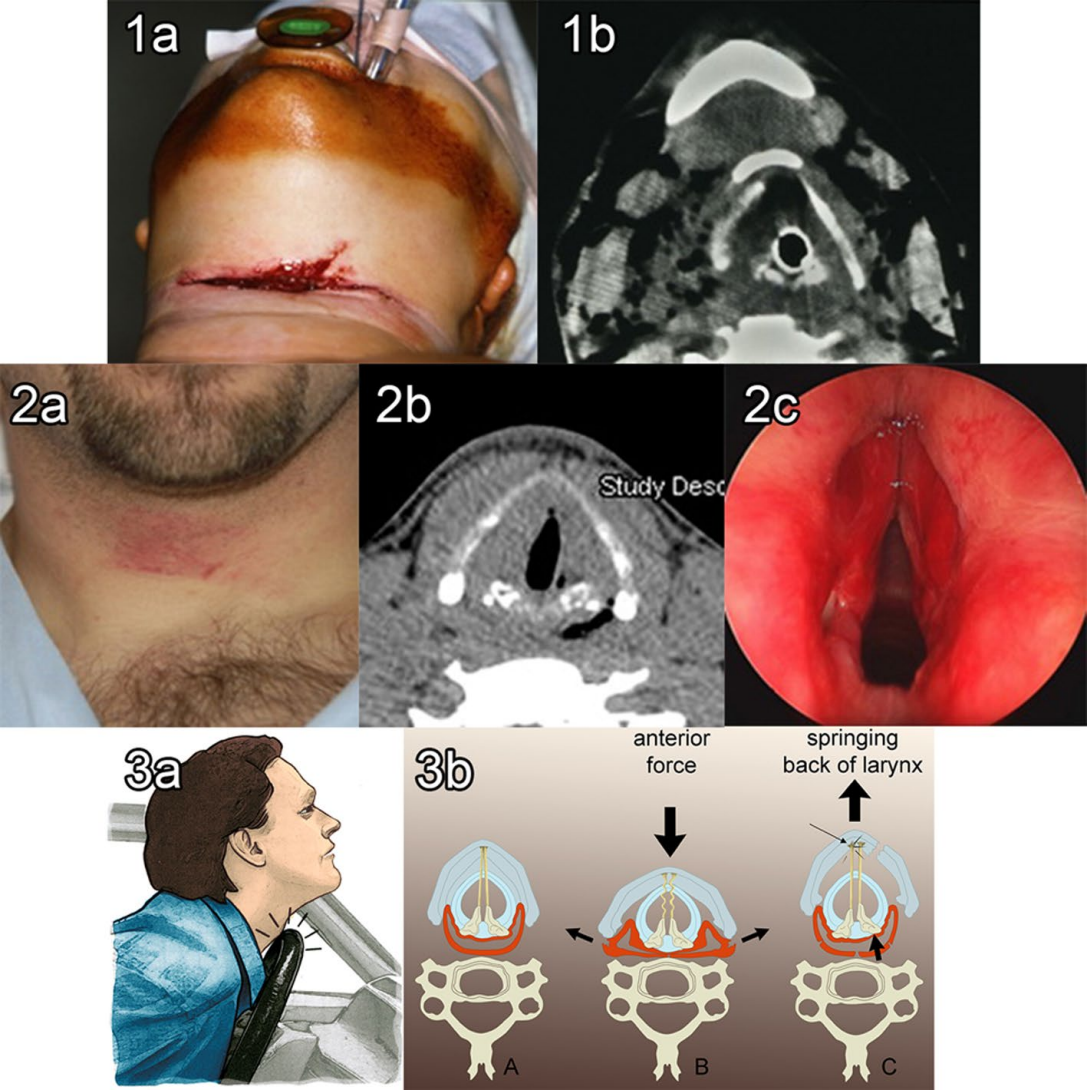
Types of laryngeal injuries. **1a** Penetrating injury. **1b** CT scan of the intubated patient showed a thyroid cartilage fracture. **2a** Blunt trauma injury following a hockey puck injury. **2b** CT scan showed an air pocket in the right retro-arytenoid region. **2c** Bilateral vocal cords hematoma. **3**. Mechanism of blunt laryngeal injury. **3a** Crushing forces on the anterior neck due to the steering wheel during deceleration with hyperextension of the neck. **3b** [A] Vocal cords and the pharynx in resting state. [B] The anterior force causes median and paramedian thyroid cartilage fractures and pharyngeal lacerations (black arrows). [C] Springing back of the larynx causes arytenoid dislocation and vocal cord(s) disinsertion at the anterior commissure

External laryngotracheal trauma (ELT) includes blunt and penetrating injuries (Fig. 1). Studies estimate an incidence of 1/14000–1/40000 emergency visits with a mortality rate as high as 40% from blunt injuries and 7–20% from penetrating injuries [1–5]. The immediate mortality rate is even higher (80%) when the thoracic trachea is involved because of the associated vascular injuries and when the head and neck or other adjacent body sites have an injury [6, 7].

Due to increased diagnostic imaging and better aero-digestive endoscopy tools, a recent study shows higher incidence rate of ELT in 1/1042 hospital admissions and in 1/2478 emergency presentations with a mean patient age being 40 years[4]. Concomitant injuries along with laryngotracheal trauma include cranio-cerebral trauma (13%), open neck (9%), cervical spine (8%) and pharyngeal or esophageal injuries (3%) [2, 6].

Motor vehicle accidents cause ELT in 60% of the cases (car 37%, motorcycle 23%) by assault or suicide attempts in 20% ( knife wounds or hanging) and by accidental blows to the neck during contact sports (karate, wrestling, soccer, ice hockey) or occupational accidents from rotating blades, or by falls (sky, mountain bike) in the remaining 20% [3, 4]. During peacetime, blunt injuries are more common (83%) compared with penetrating injuries (17%), and associated thermal burns during ballistic war injuries add to the complexity of the trauma [7, 9].

Because of the high localization in the neck, the pediatric larynx is well protected and is not commonly injured, though play activities (fall from a height, sports accidents) can cause these injuries in children.

At our tertiary university hospital setup, with a well-established emergency service, difficult airway response team (DART), and a trauma center, we have referred 2–4 cases of ELT with varying degrees of complexity each year. In this report, we look at the specific types of laryngotracheal injuries, review the various treatment protocols for managing a patient with ELT, and propose an easy-to-follow treatment algorithm.

### Ethical statement

In preparation of this report, the authors complied with all guidelines of the Committee on Publication Ethics.

## Methods

After obtaining institutional board review approval and local ethics committee authorization (CER-VD 2020), records of all patients were obtained from our hospital’s electronic charts and analyzed. We studied 22 patients with ELT treated at our center from January 2005 up to December 2021 with varying grades of injury [5]. All patients had at least one endoscopy before being discharged from the hospital. We looked at their current breathing status and their voice, and this information was obtained by emails written to the treating doctors.

### Management strategy in the emergency department

**Fig. 2:**
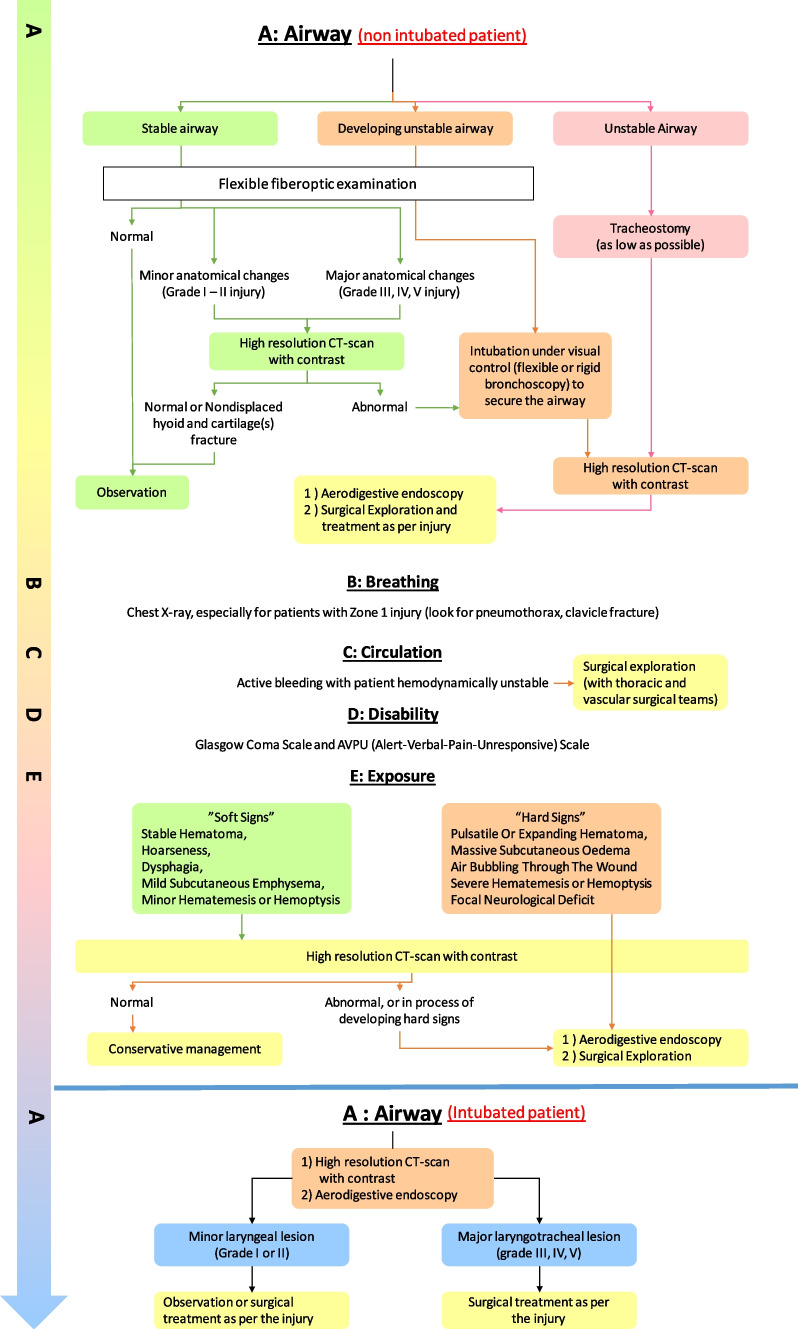
Management strategy for laryngotracheal trauma in the non-intubated and intubated patient

Figure 2 shows our unit’s protocol to manage patients with ELT presenting to the emergency room (ER) either in a non-intubated or an intubated state.

The first evaluation follows an “A, B, C, D, E” assessment in the ER, where there is a close collaboration between the difficult airway response team [8, 9] that includes emergency physicians, anesthesiologists, ENT and other services (radiology, neurosurgery, orthopedics, trauma surgery etc.).

### ABCDE assessment

A: Airway. The physical examination began by looking for an *unstable airway criteria* that included—expansive neck emphysema, air bubbling through an open wound, dyspnea with stridor, severe hematemesis or hemoptysis.

**Fig. 3:**
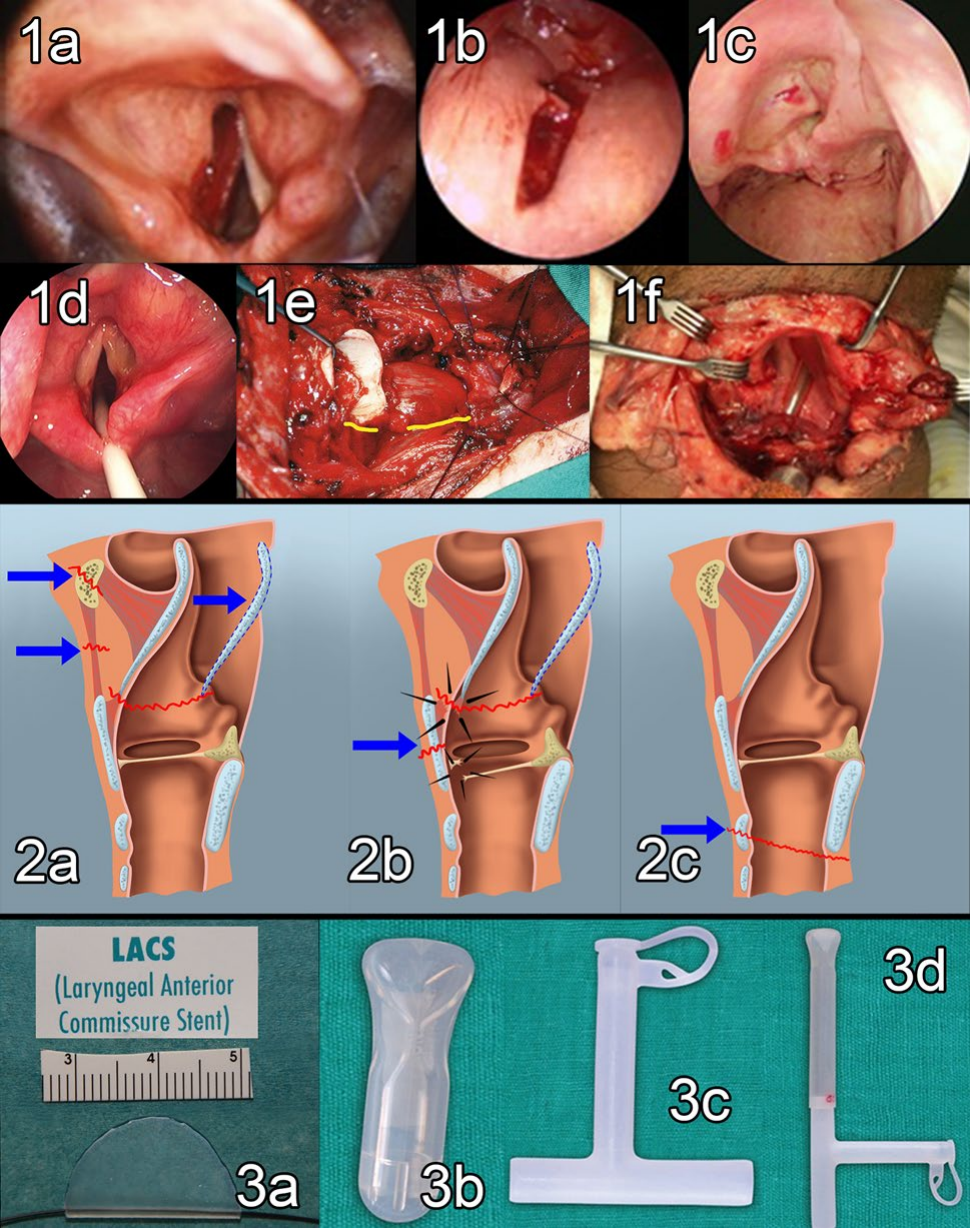
Laryngotracheal injuries. Endoscopic photos of various types of laryngo-pharyngo-tracheal injuries. **1a** Left vocal cord hematoma. **1b** Pharyngeal tear. **1c** Posterior displacement of the epiglottis following a hyoid bone fracture. **1d** Antero-medial dislocation of the right arytenoid cartilage. **1e** Laryngotracheal separation (Note the yellow marking showing the right recurrent laryngeal nerve). **1f** Severe pharyngolaryngeal substance loss following cut-throat injury (note the distal tracheostomy). Schema showing types of laryngeal injuries and their possible sequelae. **2a** Injury passing through the hyoid bone and thyrohyoid membrane causes disruption of the epiglottic petiole and can cause supraglottic stenosis. **2b** Injury passing through the thyroid cartilage and epiglottic petiole causing vocal cord disinsertion can cause supraglotto-glottic stenosis. **2c** Injury passing through the cricoid cartilage can cause a subglottic stenosis. Different types of stents are used in laryngotracheal trauma. **3a** Laryngeal anterior commissure stent (LACS) used in an anterior commissure injury. **3b** Monniers’ LT mold used in laryngotracheal injury. **3c** Montgomery T Tube used in tracheal injury. **3d** LT mold mounted on a T tube used in extensive laryngotracheal injury

Depending on the situation, intubation was done using either a flexible bronchoscope used as a guide to pass the endotracheal tube through the traumatized larynx or by using a rigid ventilating bronchoscope (Storz, Germany). Precise diagnosis of laryngeal dynamic functions and local mucosal-cartilage lesions is difficult once an endotracheal tube has been passed. Therefore, when it was judged possible, a flexible bronchoscopy followed by an examination with a Rod lens telescope was made (Fig. 3).

In the presence of *complex ELT*, a tracheostomy was done after securing the airway with an intubation or after having a rigid ventilating bronchoscope in place. We prefer doing a distal tracheostomy or sometimes passing the tracheotomy cannula directly through the exposed airway (in case of a penetrating injury) and confirming its placement in the distal airway with a thin, flexible bronchoscope passed through the cannula.

In the case of a *stable airway*, a flexible nasofibroscopy provided much information. We looked for upper airway bleeding, laryngeal mucosal lesions and oedema, vocal cord functions and accessibility of the airway. This subjective evaluation also determined the intubation protocol. In case of impossible oral access (due to excessive swelling or disrupted facial bones), a tracheostomy was performed after the airway was secured.

In case of *major anatomical modifications in the neck* secondary to subcutaneous emphysema or expanding hematoma, we preferred intubation with the patient breathing spontaneously using flexible endoscopic guidance. In a similar situation sometimes, an awake intubation could be an attractive option but this requires collaboration of the patient. Topical anesthesia in the upper airway in both techniques is crucial. In the context of *cervical spine trauma*—sedation, maintaining spontaneous breathing, neck immobilization and flexible bronchoscopy-guided intubation is ideal.

In a patient who was *stable in the emergency ward, but progressively develops an acute airway obstruction*, prior close examination of the vocal cords and subglottis is critical. This could avoid worsening a distal airway injury or even losing the airway during the intubation (in case of a cricotracheal separation).

In a patient with a stable airway, having no airway symptoms and a normal nasofibroscopy, the probability of an inconspicuous injury is low. In such cases, standard intubation, followed by a detailed aero-digestive examination was done.

B: Breathing: A chest X-ray was performed for patients with low cervical injuries, looking for signs of pneumo-/hemo-thorax.

C: Circulation: This point concerns ELT with a penetrating wound wherein the carotid artery, internal jugular vein, thyroid gland and vessels, other neck and mediastinal vessels may be injured. In case of hemodynamic instability due to massive bleeding, the patient was transferred directly to the operating theatre having a head-neck and vascular surgeon backup.

In a totally stable patient, a contrast CT scan and Doppler angiography were performed to know about the degree of vascular injury, and then decide the treatment strategy either by interventional radiology (via embolisation) or by open surgery.

D: Disability: Emergency physicians assessed the patient’s level of consciousness using the AVPU scale (Alert: the patient is vigilant, although not necessarily orientated., Verbal: the patient makes some kind of response when spoken to, Pain: the patient responds to a painful stimulus, Unresponsive: the patient does not show evidence of any eye, voice or motor responses to pain) [10].

E: Exposure: For a patient with ELT injury having a stable airway and was hemodynamically stable, the treating team closely examined the neck, thorax and abdomen for other injuries.

### Follow up

A significant number of patients (90%) were referred from other institutions. Prior to discharge from our hospital, all patients had a check endoscopy and were evaluated by our speech and swallow therapists.

Respiration was subjectively evaluated as normal, with forced exertion, with moderate exertion, at rest and objectively by endoscopy.

The pattern of patient referral and the fact that they were seen by different doctors after returning to their institutions made it difficult to have an optimal objective voice assessment. Phonation was judged as normal voice, mild dysphonia (described as hoarse voice with some difficulties of being heard or understood in a loud environment), moderate dysphonia (described as weak to breathy voice, easy fatigability) and severe handicap with difficulty to communicate.

## Results (Table 1 and 2)

**Table 1:** Patient details

22 Patients		
Age	63 years (range: 11y-82y)	
Sex	18 men and 4 women	
Cause of laryngotracheal trauma	*N*	%
Sport injury	6	28
Road accident	8	36
Home accident	4	18
Mob agression	2	9
Suicide	2	9
Intubation required		
On site	2	9
In intensive care while waiting for the surgery	3	14
In operating room, and not extubated after surgery	5	23
Schaefer’s classification		
1. Minor hematoma of endolarynx or non-fracture laceration	4	18
2. Extreme edema, hematoma, a fracture that is not dislocated or break in the mucosa in which cartilage is not exposed	5	23
3. Extensive edema, extensive break in the mucosa, dislocated fractures or immobility of vocal cord, exposed cartilage	6	27
4. Serious interruption of the anterior larynx, unsteady fractures, fracture lines, and severe trauma of mucosa	4	18
5. Laryngotracheal separation a. complete (2) b. partial (1)	3	14
Tracheostomy before management at our center	8	36
Radiologic findings		
Hyoid bone fracture	1	5
Thyroid cartilage fracture	9	41
Thyroid plus cricoid fractures	3	14
Cricotracheal separation	3	14

**Table 2:** Management and patient outcomes

Management	
No treatment (*n* = 8; 36%)	
Endoscopic procedure (*n* = 2; 9%)	
Mucosal repositioning and anterior commissure splint placement	1
LT mold insertion	1
Open surgery (*n* = 12; 55%)	
Hyoid bone fixation and epiglottic petiole positioning	1
Cartilage reduction and external fixation	8
Laryngotracheal anastomosis	3
Recurrent nerve exploration	0
Management of cataclysmic bleeding	0

Outcomes		
Death	None	0%
Tracheostomy		
Long term/permanent tracheostomy	0	0%
Time to decannulation	92 days (range 62d-138d)	
Voice		
Normal	16	73%
Dysphonia (mild/moderate/severe)	6 (4/2/0)	27%
Vocal cord palsy		
Complete recovery	3 (/7 unilateral), 1(/1 bilateral)	
No recovery	4 (/7 unilateral)	
Dysphagia		
Oral feeds (OF) *without* aspiration (A)	15	68%
OF *with* A and complete improvement in < 3 months	7	32%
Permanent enteral feeding	0	

Our 22 patients included 18 men and 4 women, with a median age of 63 years (range: 11y-82y). Most patients had sports trauma and roadside or accidents at home. The various Schaefer’s grades of injuries are summarized in Table 1. Eight patients had vocal fold immobility (7 unilateral, 1 bilateral). More than half of the patients had severe grades (Schaefer 3–5) of laryngotracheal injuries. Trauma in all patients was limited between the hyoid and upper trachea, without causing any major neurovascular injury.

Eight patients had tracheostomy prior to coming to our center and 10 patients were intubated (2 on-site of trauma, 3 in the intensive care while waiting for surgery, and 5 in the OR).

Radiology mainly showed laryngotracheal injuries and no major cranio-spinal and thoraco-abdominal trauma needing urgent attention.

Two patients (9%) were treated endoscopically, 12 (55%) had open surgery and 8 (36%) were only closely observed. Of the patients who had open surgery, 1 had hyoid bone fracture and needed epiglottic petiole positioning, 9 had thyroid cartilage fracture, 3 had thyroid plus cricoid fractures and 3 had cricotracheal separation (2 complete and 1 partial). Recurrent laryngeal nerve was not explored in any patient. There were no deaths.

At the last follow-up, all eight patients were decannulated within a median period of 92 days (range 62d-138d) and had normal respiration.

Sixteen patients had normal voice, and 6 continue to have mild to moderate dysphonia. 3 out of 7 patients with unilateral vocal cord palsy and one with bilateral nerve damage had complete recovery. No patient required medialization laryngoplasty to improve voice and/ or swallowing.

All patients achieved normal oral feeding within 3 months, and none required long-term or permanent enteral feeding.

## Discussion

The cornerstone in the treatment of an ELT is a prompt initial diagnosis, securing the airway and management of the laryngotracheal and pharyngoesophaeal injuries to avoid both—immediate fatal respiratory distress and catastrophic long-term aero-digestive sequelae.

Over the years, data collected by various centers have permitted improvement of the diagnostic strategies amid initial management through established protocols and provided clear indications for conservative and surgical treatment based on the type of injury.

Our airway unit developed a practical algorithmic approach (Fig. 2) to manage ELT trauma, and in our opinion following are some practical tips for a successful outcome while managing these difficult injuries:

### Zone-wise management

Various authors tried to define neck zones to help determine the nature and degree of tissue injury [7]. Zone I is below the omohyoid muscle corresponds to the supra- and infra-clavicular areas and may involve the innominate vessels, common carotid artery, subclavian vessels, vertebral artery, brachial plexus, trachea, oesophagus, lung apex, and thoracic duct. Zone II is between the hyoid bone and the cricoid cartilage and includes the thyroid gland, carotid and vertebral arteries, internal jugular veins, trachea, and oesophagus. Finally, zone III is above the hyoid bone and up to the base of the skull. Trauma in this zone will implicate injury to the proximal carotid artery, the vertebral arteries, and the pharynx.

All our patients had injuries in zone II, predominantly involving the larynx and trachea and none injuring the major neck vessels and nerves, the pharynx and the oesophagus.

Recently, most authors advocate a “no zone management” [11, 12] and recommend searching for signs that correspond to the underlying injury. “Major or Hard signs” include pulsatile hematoma, expanding hematoma, massive subcutaneous oedema, air bubbling through the wound, severe hematemesis or hemoptysis, or focal neurological deficit. “Minor or Soft signs” include stable hematoma, hoarseness, dysphagia, mild subcutaneous emphysema, minor hematemesis or hemoptysis, neck pain, cough, change of voice, and aspiration (that may appear later when the patients begin to eat).

### Tracheostomy in patients with ELT

In the case of complex ELT, all the usual anatomical landmarks in the neck could be lost or hidden by a hematoma, subcutaneous oedema/emphysema, or a laryngotracheal separation. In such cases, it is best to start with a flexible bronchoscopy in the patient breathing spontaneously to gather maximum information about the airway proximal and distal to the trauma site, secure the airway by intubation or by rigid bronchoscopy and then perform the tracheostomy. In our opinion, a surgical cricothyroidotomy must be avoided as it adds to the airway trauma, and it anyway gets converted to a tracheostomy during surgical exploration. Tracheotomy under local anesthesia is risky in cases of lost anatomical landmarks, and may evoke a sudden deterioration of the airway.

The new tracheostomy is a source of infection to the laryngeal injury and must be placed further distally, depending on the extent and site of the LT injury. A prior high tracheostomy should be placed more distally during surgical correction of the injury.

### Radiology

The decision about the appropriate timing and the indication of a CT scan (high-resolution of the head, neck and thorax with contrast media injection) is based on the patient’s overall stability. For a stable or a stabilized patient, the imaging will help the surgery or decision-making in a conservative approach. Most authors [4, 7, 12] recommend that patients with ‘hard signs’ must have an immediate CT scan (after securing the airway) before going to the operating room to detect the source of bleeding, hematoma, subcutaneous emphysema, pneumo ± hemothorax and the potential laryngeal lesions (fractures or dislocations of the cricoid, hyoid, thyroid cartilage, arytenoid luxation). However, the patient must be rushed to the operation theatre without a scan in case of torrential bleeding with a backup of vascular surgeons. The CT scan in a patient with ‘soft signs’ will help to determine whether a surgical exploration is required.

### Management in the operating room

The goal of surgical management is to re-establish airway patency and preserve or recreate the functional anatomy of the upper aero-digestive tract.

### Endoscopy

The main objectives of performing an upper aero-digestive endoscopy are: (1) evaluate the dynamic functions of the upper airway; (2) systematically document the airway lesions (pharynx, larynx, trachea and bronchi); (3) secure the airway; (4) perform a pharyngo-esophagoscopy to rule out pharyngeal tears, oesophageal trauma and insert a nasogastric tube.

In our experience, injuries in the pharynx, pyriform sinus and upper oesophagus are best diagnosed by carefully using a rigid telescope and without further traumatising the injured mucosa. Flexible endoscopy causes collapse of the pharyngeal mucosa and minor injuries may be missed. Additionally, insufflation of the upper digestive tract during flexible pharyngo-eso-gastroscopy can cause subcutaneous emphysema, pneumomediastinum and pneumothorax.

### Management of individual airway injuries (Fig. 4)

**Fig. 4:**
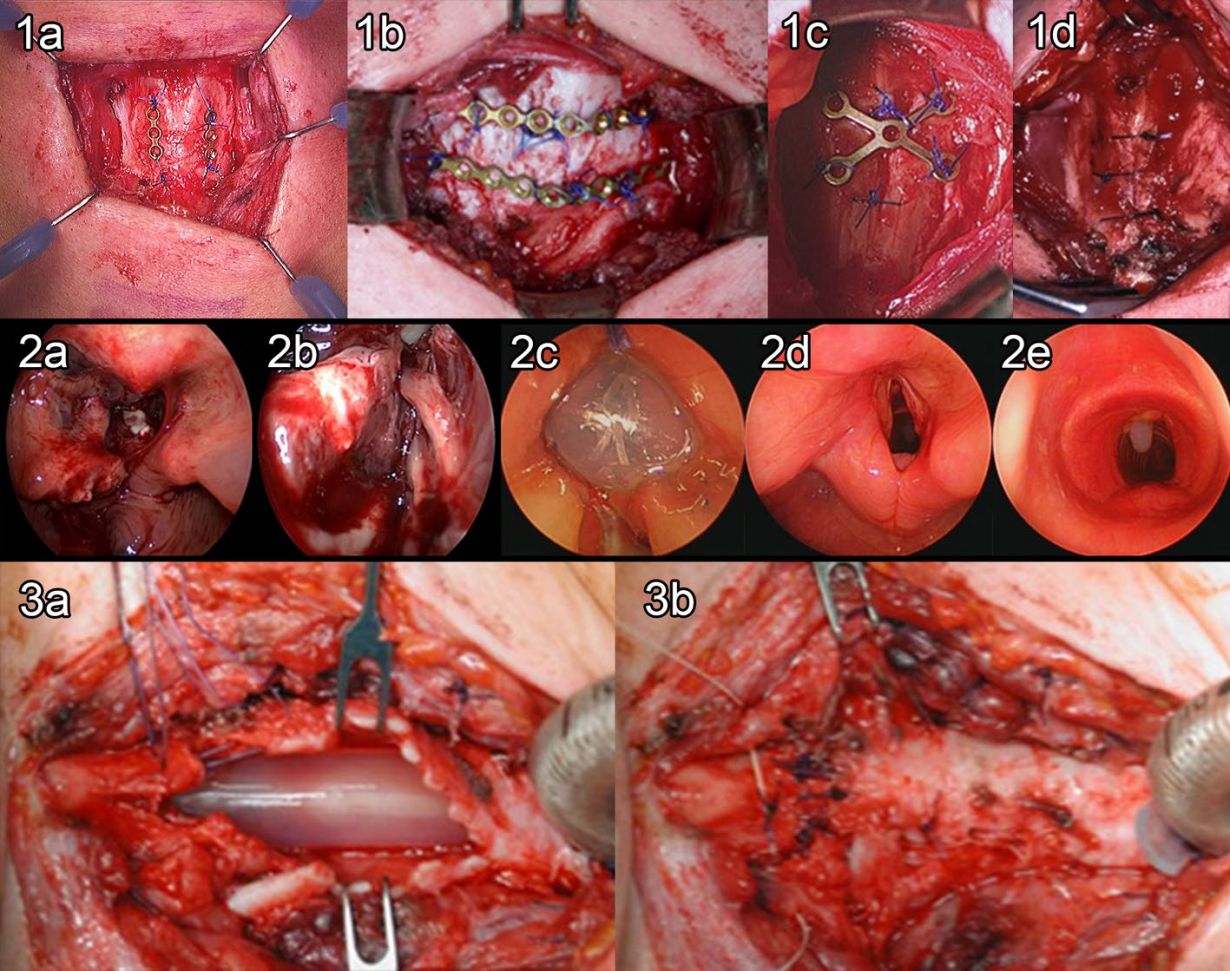
Surgical repair of LT injuries. **1a** Metallic mini-plates used in paramedian *longitudinal* thyroid cartilage fracture. Miniplates are fixed using either non-resorbable Prolene sutures or metal screws. **1b** Miniplates for a *horizontal/ transverse* thyroid cartilage fracture (Note: horizontal fractures of the thyroid cartilage are rare). **1c** Cruciate shape mini-plate fixed with 3.0 Prolene in a paramedian fracture. **1d** Resorbable 3.0 Vicryl sutures stabilizing a median thyroid cartilage fracture. **2a**, **2b** Downhill cycling injury causing severe penetrating glotto-subglottic trauma with severe laryngeal laceration and rupture of both cricoarytenoid joints. **2c** The patient underwent a complex laryngotracheal repair and insertion of LT mold. **2d**, **2e** Optimal anatomical and functional results were obtained and the patient was successfully decannulated. **3a**, **3b** Schaefer grade V injury showing torn tracheal cartilages. Note the distal tracheostomy and repair of the injury using an LT mold. The patient had a successful post-operative result.

*Hyoid bone fracture*: Hyoid fractures are caused mainly by strangulation or hanging.

Isolated fracture of the hyoid bone is usually treated conservatively with diet changes and analgesics, as it is painful during swallowing. In the presence of displaced or comminuted fractures, it is critical to realign and fix the osseous fragments using mini-plates (titanium or biodegradable polymers). In addition, the petiole of the epiglottis must be fixed in the correct position to prevent supraglottic stenosis in the future.

One of our patients needed an additional endolaryngeal stenting using the LT mold (Lausanne University Hospital, CHUV, Switzerland, Fig. 3, 3b) to prevent collapse of the supraglottic airway and required a temporary distal tracheostomy.

Supraglottic injuries: Exploration of the supraglottic area is made through the thyrohyoid membrane. Disruption of the attachment of the epiglottis is treated by anterior fixation of the petiole with the thyroid cartilage using resorbable sutures and an LT mold to support the endoluminal collapsing tissues and promote optimal healing. Significant injuries of the supraglottic area with extensive lacerations are managed with a supraglottic laryngectomy, as performed for oncologic purposes.

### Cartilage fractures

External blunt trauma compresses the thyroid, cricoid and extra-thoracic tracheal cartilages against the spine (Figs. 1, 3a, b). Damage to these cartilages depends on the degree of calcification, meaning the injuries will be worse in elderly patients. A blunt injury typically results in a vertical, linear, median, or paramedian fracture. Special care must be given in these cases during the dynamic endoscopy because the incidence of concomitant uni- or bilateral recurrent laryngeal nerve palsy(s) is high. The rebound impact on the thyroid cartilage disrupts the anterior glottic commissure and the epiglottic petiole insertion, and if left untreated or is sub-optimally treated can cause supraglottic and glottic stenosis (Figs. 2a, b, c, 3).

Exposed cartilages, multiple or displaced cartilaginous fractures require open surgical exploration. Fragments must be reduced and fixed with titanium osteosynthesis miniplate(s) (DePuy Synthes, Zuchwil, Switzerland) (Fig. 4), stainless-steel wire, or non-absorbable monofilament sutures (3, 4.0 Prolene, Ethicon). Patients with crushed cricoid ring will require a tracheostomy. Vertical cricoid fractures can be fixed with mini plates and temporary LT mold stabilization. Select non-displaced fractures may be managed conservatively and could have a good prognosis.

### Arytenoid dislocation

It is preferentially treated endoscopically, except by an open surgery in the presence of other injuries. Isolated arytenoid dislocation has good functional results if they are reduced within three weeks [4], followed by speech therapy. Sometimes, cricoarytenoid ankylosis (CAA) may subsequently develop, and the patient will require a long-term follow-up. In the case of a unilateral joint fixation, contralateral cord mobility may compensate, and by itself may not cause long-term respiratory distress. However, sometimes to improve phonation and/or swallowing, overcompensation by redundant supra-arytenoid mucosa on the side of the fixity in uni- or bilateral CAA can occur causing respiratory symptoms and this may need limited laser trimming of the floppy tissue. In extreme cases of bilateral fixation, posterior arytenoidectomy or a laryngofissure with posterior glottic expansion using a rib cartilage graft will be necessary to treat glottic obstruction.

### Glottic injuries

Severe damage will require a tracheostomy if it has not been already done in an emergency. Mucosal lacerations at the anterior commissure will require a laryngeal Keel placement (Laryngeal Anterior Commissure Stent LACS, Boston Medical Products) (Fig. 3a). Massive mucosal lesions will require an LT mold placement to avoid a future dense glottic synechiae formation and vocal cords fusion. Both, the LACS and the LT Mold can be endoscopically fixed in the glottis under suspension laryngoscopy using a Lichtenberger’s needle carrier [14] and 3.0 Prolene suture.

### Subglottic injury

A laryngotracheal disruption is one of the most severe laryngeal injuries. It is nearly always associated with uni- or bilateral recurrent laryngeal nerve injury(s). In addition, concomitant cricoid fractures and pharyngoesophageal tears are often present.

Meticulous debridement, correct approximation of the laryngeal and tracheal stumps, and tension-free anastomosis are the keys to a successful laryngotracheal reconstruction. Associated cricoid fractures are reduced and fixed with resorbable (Vicryl or polydiaxanone) sutures or osteosynthesis titanium mini plates. Internal stabilization with an LT mold may be necessary in the presence of extensive damage to the laryngotracheal framework and fractures of the posterior cricoid plate.

Multilevel LT injuries may require a Montgomery T tube or the LT mold placed over a T tube.

Missed phayrngo-esophageal injuries might be left untreated, and may cause aero-digestive fistula and mediastinitis with catastrophic consequences.

### Indications for airway stenting [4]

We stent an ELT in the following situations:

- Disruption of the anterior half of the larynx
- Extreme instability of the LT framework, which cannot be maintained with external fixation of the cartilaginous fractures
- Severe loss of the cricoid structural integrity
- Massive endolaryngeal mucosal laceration and avulsion with a high risk for cicatricial stenosis
- Extensive lacerations of the mucosa, preventing the restoration of a normally shaped anterior commissure

### Indications for recurrent nerve exploration

In patients with laryngotracheal disruption or cricoid fracture, recurrent nerve(s) damage is frequent [4]. Systematic nerve exploration is not recommended as it may be difficult in swollen tissues and could lead to further damage to a potentially intact nerve. If the nerve transection is obvious, a direct end-to-end anastomosis can be attempted after freshening the nerve edges or recurrent to ansa cervicalis anastomosis may be performed [4].

The main drawback of this communication is its retrospective design applied to a small number of patients with rare injuries.

## Conclusion

The success of managing external laryngeal trauma relies on fast decision-making, correct patient evaluation, securing the airway and maintaining hemodynamic stability. Close collaboration among the emergency physicians and the ENT surgeons is crucial, and the team members must know their role, the resources available and their individual limitations.

Management of cerebral, spinal and vascular injuries associated with laryngeal trauma takes precedence, and aero-digestive injuries can be forgotten in the early hours of management. Missing out to diagnose these injuries is a big error in the management strategy and will have disastrous consequences. Endoscopy is the gold standard in the diagnosis of aero-digestive injuries. These injuries can be compared to open limb injuries where every minute counts, and failure to provide prompt treatment can leave the patient severely handicapped for life.
